# Masturbation in a male Phayre's langur, *Trachypithecus phayrei*

**DOI:** 10.5194/pb-8-43-2021

**Published:** 2021-11-18

**Authors:** Md Shalauddin, Md Jayedul Islam, Tanvir Ahmed

**Affiliations:** Department of Zoology, Jagannath University, Dhaka 1100, Bangladesh

## Abstract

Masturbation is a common auto-sexual behaviour in humans but is not explicitly known in a major portion of non-human primates. We report the first masturbatory behaviour in a male Phayre's langur, *Trachypithecus phayrei*, observed in a semi-evergreen forest in northeastern Bangladesh. Like other Asian colobines, the multi-male–multi-female groups of the Phayre's langur suggest a multilevel social organization and a complex hierarchy among males. The lack of sexual opportunity could result in masturbation and sperm competition among males. However, sperm competition is reported to be low in such non-seasonal breeders. Hence, we suggest an adequate characterization of the socio-sexual behaviour and reproductive strategies of this globally endangered primate in order to demonstrate the causes, cost and consequences of masturbation. We also urge further scientific exploration into masturbation among primates due to its evolutionary and conservational significance.

Masturbation, a common auto-sexual behaviour in humans, has been considered a taboo, unnatural pursuit or even a pathological behaviour for centuries (Laqueur, 2004; Thomsen and Sommer, 2015). Similar to the modern human, masturbation has been reported in both sexes of non-human primates, and masturbatory behaviour differs between sexes. Males use their hands, feet or mouth to stimulate their genitals until ejaculation occurs, whereas females exhibit extensive sophisticated behaviours including stimulation of the anogenital and breast regions using various “tool-like” substrates (Thomsen and Sommer, 2015). However, masturbatory behaviour is not explicitly known in a major portion of primates (approximately 80 % of primates; inferred from Thomsen and Sommer, 2015), perhaps due to the illusive nature of masturbatory events and the current lack of studies on the topic (Thomsen et al., 2003).

Phayre's langur, *Trachypithecus phayrei* (Blyth, 1847), is a globally endangered colobine primate, distributed from eastern Bangladesh, through the Assam, Mizoram and Tripura states of northeastern India, and into the west of the Chindwin and Ayeyarwady rivers in western Myanmar (Chetry and Ahmed, 2021; Roos et al., 2020). This predominantly folivorous primate lives in multi-male–multi-female groups, and the group sizes range from 4 to 26 individuals (Ahmed et al., 2020). It is one of the least studied primates in terms of ecology, behaviour, genetics and systematics (Roos et al., 2020). Here, we report an observed masturbation event involving a male Phayre's langur in Lawachara National Park, a 1260 ha semi-evergreen forest in northeastern Bangladesh.

**Figure 1:**
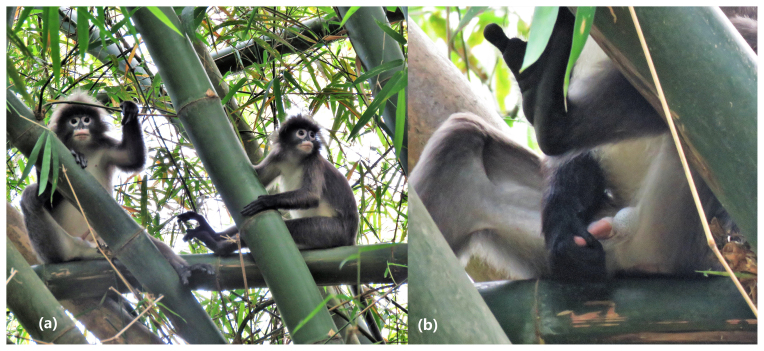
Two Phayre's langur individuals (left: M2; right: M3) resting in Lawachara National Park, Bangladesh: **(a)** the erect penis of M2 before masturbation and **(b)** M2 rubbing the penis body with his right hand.

From February to April 2017, we followed a Phayre's langur group named “PL1” in Lawachara National Park as part of our habituation process for a year-long study on their behavioural and feeding ecology. The group was composed of 19 individuals: 3 adult males, 5 adult females and 11 non-adults including 2 infants. PL1 was not very shy in the presence of humans except for during very close encounters. On 8 April 2017, we followed the group from 07:30 to 16:00 (GMT + 6, Bangladesh time) during which time they mainly fed on leaves of different plant species and took two brief rests. The second resting period was from (approx.) 14:10 to 15:30 LT during which time the animals were seated sparsely between 3.5 and 14 m above the ground in dense vegetation close to a railway into the forest. We could only identify six individuals based on external morphological traits from our observation point. Of these individuals, two adult males “M2” and “M3” were seated on reclined bamboo (Fig. 1). At 14:08 LT, M3 slept holding a vertical bamboo, whereas M2 presented an erect penis. About 4 min later, M2 started rubbing the glans and body of his penis using the thumb and finger of his right hand and continued to do so for more than 2 min. Using a pair of Bushnell 8 × 42 binoculars, we observed ejaculation: a semi-transparent white fluid dropped out onto the forest floor. The erect organ took more 3 min to return to its usual size after ejaculation. M3 was awake while M2 masturbated and observed events for a while but did not show any reaction. We did not visit the forest floor to find the ejaculate, as this could have disrupted the habituation process.

To our knowledge, this is the first report of masturbation in Phayre's langur. Masturbation has been observed in a number of Asian colobines in the past, including 22 species of the *Trachypithecus* genus; masturbation has been reported in *T. cristatus* (Bernstein, 1968), *T. poliocephalus* (Hendershott et al., 2018) and *T. francoisi* (Hu, 2007) in the wild. Masturbation has mostly been observed in males, accompanied by facial, vocal and genital erection displays toward females. Hendershott et al. (2018) observed masturbation in a *T. poliocephalus* male when a female individual was grooming the masturbating male, but no mounting occurred. In fact, males from around 80 species and females from over 50 species from all major radiations of the living anthropoid primates are known to exhibit masturbatory behaviours (Thomsen et al., 2003; Jones, 2005). Hence, it has been suggested to be an ancestral, phylogenetically widespread trait and a facet of our own hominin ancestor's sexual repertoire (Thomsen et al., 2003).

Masturbation may have evolved to sire offspring from the coincidence of sexual desire and lack of sexual opportunity. It can lead to a hormonally induced relaxation that reduces aggression; thus, it might be of importance in primate species that live in social groups (Thomsen and Sommer, 2015). Moreover, female primates are thought to benefit from masturbation in the following ways: maintenance of genital health, avoidance of sexually transmitted diseases, attraction of males, enhancement of pheromone production and the manipulation of inseminated sperm. However, these evolutionary hypotheses still lack empirical evidence (Thomsen and Sommer, 2015). To a primate male, masturbation is costly, and the ejaculate is wasted on a large scale (Thomsen, 2006). Nevertheless, masturbation might be beneficial with respect to severe sperm competition (Baker and Bellis, 1993) by increasing the chances of fertilizing the egg when mating with a newly fertile female: masturbation in males allows for the production of new sperm with enhanced fitness by flushing out old and low-quality sperm from the genital tract (Thomsen and Sommer, 2015).

Masturbation has been reported to be considerably higher in species with multi-male–multi-female group structures (Thomsen et al., 2003). Like other Asian colobines, the multi-male–multi-female groups in the Phayre's langur suggest a multilevel social organization and a complex hierarchy among the males (inferred from Ahmed et al., 2020). In some species, males assess the risk and intensity of sperm competition during the breeding season by paying attention to the odours of other males on females or in the immediate environment of a female (Johnston and delBarco-Trillo, 2009). Given that the risk of sperm competition is lower in unmated females than in recently mated females, males are generally expected to show a preference for unmated females (Johnston and delBarco-Trillo, 2009). From our single observation, we cannot establish the risk of sperm competition in Phayre's langur males at our study site. However, sperm competition is known to be rather low in non-seasonal breeders (Thomsen et al., 2003; Jones, 2005), and the Phayre's langur is not well known to be a seasonal breeder. Hence, an adequate characterization of the socio-sexual behaviour and reproductive strategies of the Phayre's langur will be useful to demonstrate the causes, cost and intensity of masturbation in these animals. Such studies would also contribute to breeding programmes in captivity and help improve conservation tactics for small populations of these animals in fragmented landscapes. In conclusion, knowledge on masturbatory behaviours and their causes and consequences in wild non-human primates is poor; this calls for further scientific exploration due to its evolutionary and conservational significance.

## Data Availability

There are no further data apart from the observations reported in the paper.
